# Self-help therapy and recovery in psychosis: methodological considerations and service user involvement in a partially randomised preference trial

**DOI:** 10.1186/1745-6215-12-S1-A84

**Published:** 2011-12-13

**Authors:** Samantha Hartley, Gillian Haddock

**Affiliations:** 1 Psychology Services, Greater Manchester West Mental Health NHS Foundation Trust, Prestwich, UK; 2 Division of Clinical Psychology, University of Manchester, Manchester, UK

## 

A new Recovery Guide for people with experience of psychosis has been developed (Barrowclough et al, 2009), drawing on existing cognitive behavioural interventions and founded on collaborations between service users, psychologists, researchers and healthcare professionals. This poster showcases the involvement of ‘experts by experience’ in the design, delivery and evaluation of this Recovery Guide, and the therapeutic structure that supports its use. The project has input from service users by various methods, including service user researchers, a service user reference group and service user consultants. Previous work has highlighted the strong and rational preferences that people experiencing psychosis hold about their potential treatment options (Gerrard et al, 2009); service users were presented with detailed information about the Recovery Guide and about different levels of therapeutic intervention to support it (low support= telephone cognitive behavioural therapy; high support= as low support, plus group sessions facilitated by a cognitive behavioural therapist and service user researcher). Findings revealed that most would be willing to take part in a trial of psychological treatments, whether as participants receiving extra therapeutic support or receiving treatment as usual only and assisting with the progress of the research. Participants also held strong feelings about which option they would prefer to receive. Participant preferences of this kind have not been taken into account in previous psychological treatment trials for psychosis. The poster describes the trial currently being implemented to evaluate the Recovery Guide; STAR-T (Self-help Therapy and Recovery-Trial), is an NIHR-funded preference trial in which participants can elect to join their treatment option of choice, or be randomly allocated to a condition. The trial emphasises the value of choice for people experiencing psychosis, and the poster will also discuss the methodological considerations necessary to support such a preference trial. The trial also benefits from the involvement of two service user researchers, who have personal experience of psychosis, and a panel of service user consultants. The ways in which this input has helped shape the design, implementation and evaluation of the trial will be outlined.

